# Tetraspanins as Organizers of Antigen-Presenting Cell Function

**DOI:** 10.3389/fimmu.2018.01074

**Published:** 2018-05-23

**Authors:** Maria Laura Saiz, Vera Rocha-Perugini, Francisco Sánchez-Madrid

**Affiliations:** ^1^Servicio de Inmunología, Hospital de la Princesa, Instituto de Investigación Sanitaria La Princesa, Madrid, Spain; ^2^Vascular Pathophysiology Research Area, Centro Nacional de Investigaciones Cardiovasculares, Madrid, Spain; ^3^CIBER Cardiovascular, Madrid, Spain

**Keywords:** tetraspanins, tetraspanin-enriched microdomains, antigen-presenting cells, immune receptors, cell migration, antigen presentation

## Abstract

Professional antigen-presenting cells (APCs) include dendritic cells, monocytes, and B cells. APCs internalize and process antigens, producing immunogenic peptides that enable antigen presentation to T lymphocytes, which provide the signals that trigger T-cell activation, proliferation, and differentiation, and lead to adaptive immune responses. After detection of microbial antigens through pattern recognition receptors (PRRs), APCs migrate to secondary lymphoid organs where antigen presentation to T lymphocytes takes place. Tetraspanins are membrane proteins that organize specialized membrane platforms, called tetraspanin-enriched microdomains, which integrate membrane receptors, like PRR and major histocompatibility complex class II (MHC-II), adhesion proteins, and signaling molecules. Importantly, through the modulation of the function of their associated membrane partners, tetraspanins regulate different steps of the immune response. Several tetraspanins can positively or negatively regulate the activation threshold of immune receptors. They also play a role during migration of APCs by controlling the surface levels and spatial arrangement of adhesion molecules and their subsequent intracellular signaling. Finally, tetraspanins participate in antigen processing and are important for priming of naïve T cells through the control of T-cell co-stimulation and MHC-II-dependent antigen presentation. In this review, we discuss the role of tetraspanins in APC biology and their involvement in effective immune responses.

## Introduction

Professional antigen-presenting cells (APCs), which include dendritic cells (DCs), monocytes/macrophages, and B cells, are essential players of the immune system. Once an infection occurs, the innate immune system is stimulated, beginning the inflammation process to prevent the infection from spreading. Then, adaptive immune responses are required for the effective and specific clearance of the pathogen. This vital task lies on APCs, which operate at the interface between the innate and adaptive immunities. First, APCs detect foreign pathogens thanks to specialized receptors, known as pattern recognition receptors (PRRs). PRRs recognize conserved repeated motifs in microbial species, called pathogen associated molecular patterns (PAMPs), and enable APCs to discriminate between self and non-self (1). After engulfment of exogenous pathogens, APCs use their unique machinery to break down molecular antigens into small peptides and present a representative repertoire of these through a specialized immune receptor, namely, the major histocompatibility complex class II (MHC-II) molecule. This process triggers APC activation and maturation, with upregulation of surface expression of MHCII and co-stimulatory molecules. APC migration from peripheral tissues to secondary lymphoid organs is a key step for the generation of proper adaptive immunity, since antigen presentation to naïve T lymphocytes by APCs takes place primarily in secondary lymphoid organs (2). DCs have been extensively characterized and different subsets have been described (3, 4). Moreover, these cells precisely alternate their sentinel capacities with their antigenic presentation properties to favor antigen detection and migration, and antigen processing and presentation.

Tetraspanins belong to a family of small proteins (20–30 kDa) that contain four transmembrane regions spanning the plasma membrane. They also share other structural features: a small and a large extracellular loop with conserved residues, and short N- and C- terminal tails (5). In humans and mice, 33 tetraspanin members have been identified. These proteins are widely distributed in cells and tissues. Some of them are ubiquitous (CD81, CD82, CD9, or CD63), whereas others have a tissue-restricted expression (CD37 or CD53 in immune cells) (6). Tetraspanins do not have the characteristics of prototype membrane receptors. They have small cytoplasmic tails that lack known motifs involved in signal transduction (5), and there are only few reports claiming tetraspanin ligands (7). Instead, tetraspanins function as molecular organizers of multimolecular membrane complexes, which facilitate signal transduction processes (8). Through the association with proteins and lipids, they organize specific membrane microdomains with a particular composition and detergent-solubilization properties, conforming the so-called tetraspanin-enriched microdomains (TEMs) (9, 10). TEMs are distinct from other well-known membrane domains, like lipid rafts, caveolae, and GPI-linked protein nanodomains (10).

Early studies using biochemical approaches have shown that TEMs follow a hierarchical network of associations based on the strength of the interactions (5, 9). The first level comprises the direct and specific interaction of a tetraspanin with its protein partner and is resistant to strong detergent conditions. The second level is characterized by interactions between tetraspanins. These interactions are more labile, resistant to mild detergents, and regulated by palmitoylation. Cutting edge fluorescence microscopy techniques, as single-molecule tracking, phasorFLIM-FRET and super-resolution microscopy, have more recently demonstrated that TEM organization and composition is highly dynamic (10–14). Accordingly, several studies have suggested that TEM composition can differ between cells. Through the organization of TEMs, tetraspanins regulate the function of their associated partners, finely tuning a breadth of biological processes. They may have overlapping functions in some cases or can have unique roles or even opposing functions. Their importance for several pathological and physiological processes has been discussed in detail elsewhere (15–22).

Tetraspanins have been widely studied in the mammalian immune system, and thanks to the generation of tetraspanin knockout mice a deeper comprehension is being achieved. Interestingly, the existence of tetraspanins in the innate immune system of invertebrates and non-mammalian vertebrates has also been described. Marine gastropod mollusks show ubiquitous expression of CD63 and Tspn33, which are upregulated upon different immune stimulation challenges, like toll-like receptor (TLR) ligands, bacteria or viral infection (23). Similarly, CD9 expression is induced in lamprey fish after LPS stimulation (24), or in turtles after bacterial infection (25). CD37 expression is highly increased in Atlantic salmons after a secondary viral infection (26). Conversely, treatment with several immune stimulators downregulate CD9, CD53, and CD63 expression in leukocytes from teleost fishes (27, 28). The study of the innate defense mechanisms in non-mammalian vertebrates can give additional hints for the comprehension of vertebrate innate immunity. In mammals, tetraspanins are master regulators of APC function, mediating the crosstalk between the immunogenic environment and APCs, and the interplay between innate and adaptive immune cells.

Herein, we will review the function of tetraspanins in regulating each step of APC function: at the cellular level, by modulating clustering and trafficking of immune receptors; during the process of APC migration, and finally during MHC-II-dependent antigen presentation. We will also discuss the growing evidence on tetraspanins as markers of specific DC subsets.

### Tetraspanins, Negative Regulators of PRRs

**Figure 1:**
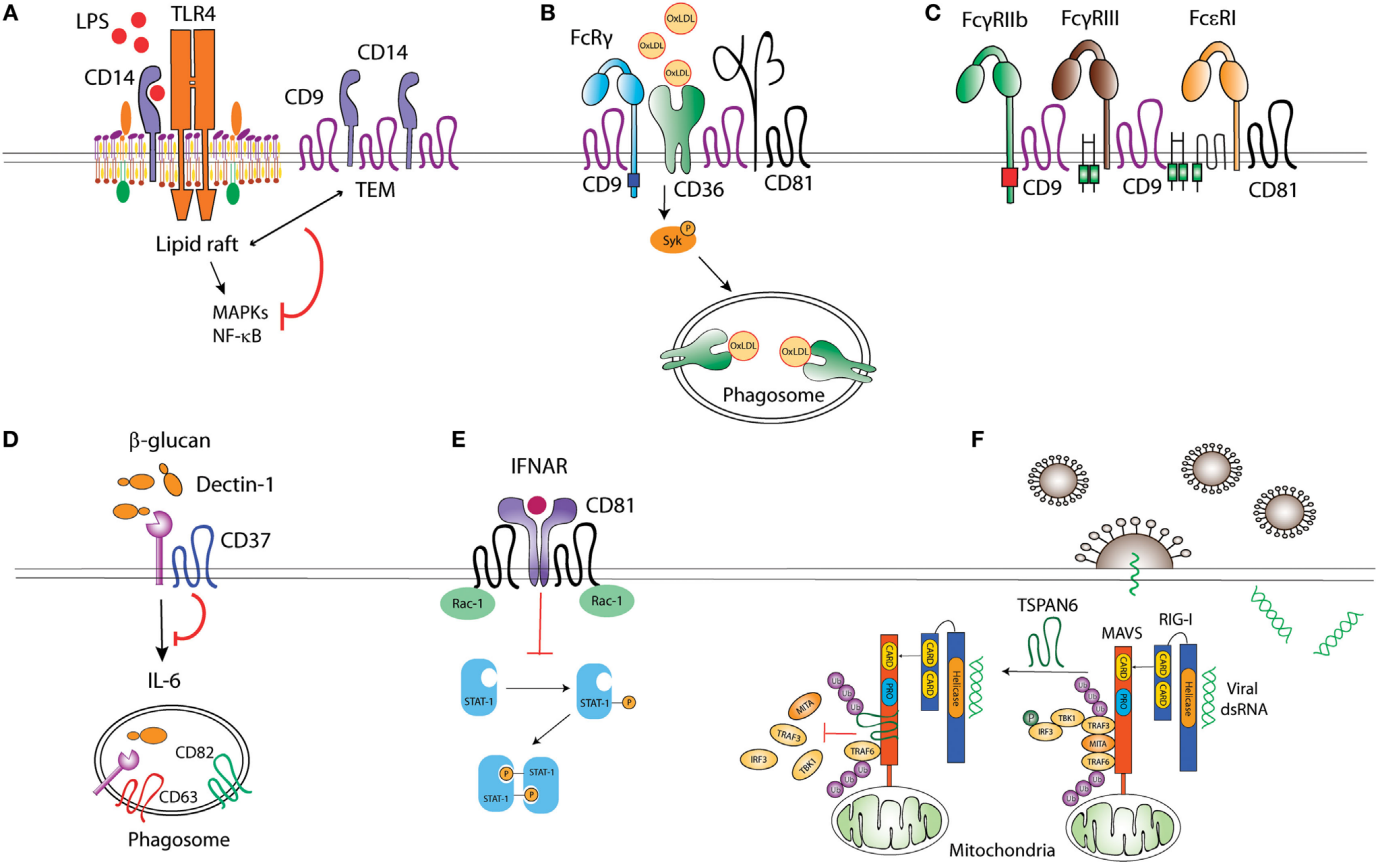
Tetraspanins act as molecular organizers of pattern recognition receptors (PRRs). **(A)** CD9 sequesters TLR4 co-receptor CD14 into tetraspanin-enriched microdomains (TEMs), impairing its localization into lipid rafts, and thus preventing TLR4 downstream signaling. **(B)** CD36 coupling to the adaptor FcRγ is mediated by the complex formed between CD36, β1, and/or β2 integrins, and CD9 and/or CD81 tetraspanins. CD36 association with tetraspanins regulates its engagement with Src and Syk kinases, and its internalization when bound to ligands like oxidized low-density lipoproteins. **(C)** CD9 collaborates with FcγRIIb and FcγRIII upon macrophage activation, and, together with CD81, associates with Fc𝜀RI. **(D)** CD37 stabilizes membrane C-type lectin receptor Dectin-1 and inhibits IL-6 production. **(E)** CD81 interaction with Rac-1 prevents STAT-1 activation downstream of interferon-α/β receptor (IFNAR) stimulation. **(F)** The interaction between ubiquitinated Tspan6 and MAVS (mitochondrial antiviral signaling) interferes with RIG-I (retinoic acid-inducible gene I)-induced recruitment of downstream molecules TRAF3, MITA, and IRF3 to MAVS after viral RNA detection.

Recognition and uptake of microbial antigens by APCs is mediated by PRRs, which bind conserved pathogen structures known as PAMPs (1). Membrane-bound PRRs include TLRs, C-type lectin receptors (CLRs), scavenger receptors (SRs) and NOD-like receptors. The efficiency of antigen recognition greatly depends on the supramolecular organization of PRRs at the APC surface, and tetraspanins play an important role in this process (Figure 1).

Toll-like receptors multimerization at the APC surface promotes the recruitment of signaling molecules (29), a process influenced by the inclusion of TLRs and associated co-receptors into TEMs. LPS stimulation triggers TLR-4 and CD81 co-clustering in peripheral blood monocytes (30). How CD81 regulates TLR-4 signaling has not been assessed; however, it has been shown that CD9 restricts LPS-induced macrophage activation and TNF-α production by preventing the TLR-4 co-receptor CD14 localization into lipid rafts (Figure 1). Through this mechanism, CD9 deficiency in mice enhances macrophage infiltration and lung inflammation after *in vivo* intranasal LPS administration (31). In DCs, bacterial antigens can be recognized by TLR-dependent pathways, sensing cell surface or endosomal antigens, and by cytosolic pathways, like the cytosolic sensor stimulator of IFN genes (STING) (32). Interestingly, CD81 negatively regulates STING/IFNAR signaling through its interaction with Rac1 and the inhibition of STAT-1 activation, thus leading to reduced TNF-α and NO production by inflammatory monocytes and DCs (Figure 1). As a consequence, CD81 deficient mice are protected against systemic *Listeria monocytogenes* infection (33).

Among the CLRs, Dectin-1 specifically recognizes β-glucans in fungal cell walls and is important for efficient immune response against fungi (34). Dectin-1 associates with tetraspanins CD37 and CD63 at the membrane of APCs when using CHAPS 1% (35, 36), a mild detergent extraction condition that only keeps third level molecular interactions within TEMs (7, 10). Dectin-1 direct association with CD37 was however observed in transfected HEK293 cells when using Triton X-100 1%, which preserves tetraspanin-partner primary complexes, but not in B cells (36), indicating that this interaction could be affected by other proteins expressed on APCs or that it is dynamically dependent on the cell activation status. CD37 stabilizes Dectin-1 surface expression and impairs its internalization, and Dectin-1-mediated TNF-α and IL-6 production in response to yeast cell walls (36) (Figure 1). Accordingly, CD37^−/−^ mice are protected against systemic *Candida albicans* infection, producing high levels of IL-6 and specific IgA antibodies (37). On the other hand, CD37 mRNA expression positively correlates with Dectin-1 and IL-6 mRNA in brains of mice infected with *Toxoplasma gondii* (38); however, further studies are necessary to evaluate this effect at the protein level and if there is any causal relationship. CD63 also seems to cooperate with Dectin-1 during yeast phagocytosis by human monocyte-derived DCs (MoDCs) (35), being specifically recruited to phagosomes containing *Cryptococcus neoformans* (39) in a process dependent on acidification and thought to be required for tethering the antigen-loading machinery together.

CD36 is a SR that recognizes proteinaceous or lipidic antigens from microbes, or self-ligands. In mouse macrophages, CD81 and CD9 are required for CD36 internalization after binding to oxidized low-density lipoprotein (oxLDL) ligands (40, 41). CD9 would be important for signaling in response to oxLDL, since oxLDL uptake and subsequent JNK phosphorylation are impaired in CD9^−/−^ macrophages (40). Moreover, CD9 and CD81-dependent scaffolding of CD36, and β1 and β2 integrins in membrane multimolecular complexes is essential for CD36 association with FcγR (Fc receptor for IgG) and with Src and Syk kinases; and for its subsequent antigen uptake (41) (Figure 1). CD9 is also associated with the scavenger-like receptor CD5, which recognizes β-glucans expressed on fungi (42), although there is no experimental evidence about the functional implications of this interaction.

Pathogens can be opsonized with IgGs produced in response to microbial invasion, and recognized by FcγRs associated with PRRs. This combined stimulation triggers cytokine production and pathogen-specific innate immune responses. FcγRs seem to be included in TEMs in phagocytic cells. CD9 antibody cross-linking, but not Fc fragment alone, stimulates intracellular signaling dependent on FcγRIIB and FcγRIII, thus promoting mouse macrophage activation (43). Antibody cross-linking of tetraspanin CD82 enhances FcR-dependent activation of intracellular signaling in human monocytic cell lines (44). Importantly, IgG-opsonized HIV-1 particles are targeted to TEMs in endosomes of immature DCs (45). Other Fc receptors are also associated with TEMs, as the FcεRI (Fc receptor for IgE), which is a molecular partner of CD9 and CD81 in human monocytes and skin-derived DCs (46) (Figure 1). The importance of TEMs as organizers of FcεRI signalosome in mast cells has been recently reviewed elsewhere (47).

Tetraspanins can also regulate signaling of cytoplasmic PRRs, like the RIG-I-like receptors (RLRs). RLRs recognize viral RNA and trigger signaling pathways that induce type I IFN responses (48). In the presence of viral RNA, ubiquitination of human tetraspanin 6 (Tspn6) promotes its interaction with RIG-I, MDA5, and mitochondrial antiviral signaling (MAVS) signalosome, impairing the activation of IFN-stimulated response element (ISRE), NF-κB, and IFN-β promoters (49) (Figure 1).

In summary, increasing evidence shows that tetraspanins usually act as negative regulators of PRR clustering and/or signaling. Thus, tetraspanins constitute key players to avoid uncontrolled immune responses, which are harmful to the host.

### Tetraspanins Tightly Control APC Migration

**Figure 2:**
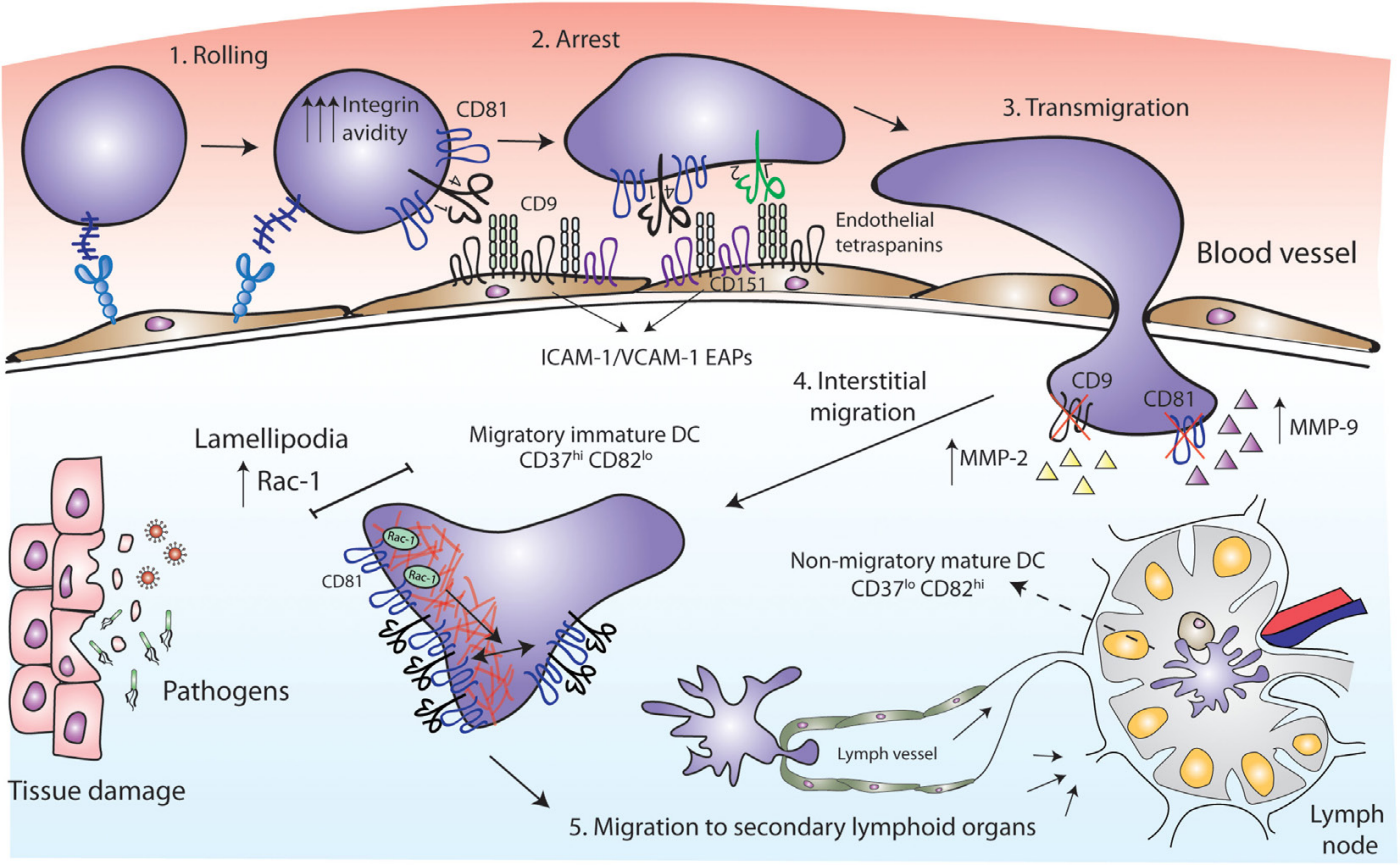
Tetraspanins act as key players in antigen-presenting cell (APC) migration. CD81 facilitates rolling and arrest under shear flow, increasing the avidity of VLA-4 integrin. Tetraspanins CD9 and CD151 congregate the endothelial adhesion receptors (ICAM-1 and VCAM-1) in clusters called endothelial adhesive platforms, thus controlling their adhesive properties and leukocyte extravasation. CD9 and CD81 deficiency results in an increase of MMP-2 and MMP-9 metalloproteinases production and activity, required for interstitial migration. Once in the tissue, CD81 tetraspanin controls cell migration *via* Rac-1-dependent mobilization of preformed integrin clusters at the leading edge and contributes to the formation of lamellipodia. While migratory immature dendritic cells (DCs) are CD37^hi^ and CD82^lo^, mature DCs at the lymph nodes are CD37^lo^ CD82^hi^, and display reduced migratory capacity and efficient antigen presentation machinery.

Leukocyte migration is of fundamental importance for the efficient development of immune responses against pathogens. Innate immune cells capture antigens in peripheral tissues and then migrate to secondary lymphoid organs where antigen presentation to T lymphocytes takes place. Immune cells can also migrate out of the bloodstream toward the inflammation site, where adaptive immune responses occur (Figure 2). Thus, leukocytes modify their adhesive properties depending on the immune scenario (50). Innate immune cells usually need inflammation signals to initiate migration, whereas naïve lymphocytes efficiently migrate to secondary lymphoid organs, and after activation signals acquire specific migratory patterns. Tetraspanins have emerged as key regulators of cell migration, since they modulate the function of proteins involved in cell-cell adhesion, cell-ECM (extracellular matrix) adhesion, cytoskeletal protrusion/contraction, and proteolytic ECM remodeling. Indeed, tetraspanins associate with integrins, cadherins, members of the Ig superfamily, signaling molecules like Rac and Rho GTPases, and matrix metalloproteinases (MMP); regulating their membrane compartmentalization, intracellular trafficking, and proteolytic activity. Most of the information on tetraspanin regulation of cell migration comes from studies with adherent and tumor cells and has been reviewed in detail (20, 51). In this section, we will delineate the importance of tetraspanins for migration and extravasation of APCs.

Early studies employed cross-linking with monoclonal antibodies (mAbs) to investigate the role of tetraspanins in immune cell migration. Human MoDC *in vitro* migration toward MIP-5 and MIP-1α chemokines was increased by the treatment with mAbs against CD9, CD63, CD81, or CD82 (35). These chemokines are strong chemoattractants required for the recruitment of inmature DCs to the surrounding tissue at the sites of injury (52, 53). After antigen capture, DCs mature, lose their responsiveness to inflammatory chemokines and express CCR7 (54, 55). CCR7 is the receptor for CCL19 and CCL21, which are chemokines highly present in lymphoid T-cell zones of secondary lymphoid organs (56), where DCs home to present their processed antigen to T lymphocytes. Opposite to that observed with MoDC migration toward MIP-5 and MIP-1α, the same mAb against CD81 (clone JS-81) or a CD81 ligand [the Hepatitis C Virus E2 envelope glycoprotein (57)] inhibited MoDC migration in response to CCL21 *in vitro* (58). These contradictory results could be due to different chemokine stimuli or to technical issues. Subsequent studies were all in line with a positive role for tetraspanins in cell migration. Monocyte transmigration across brain endothelial cell monolayers was significantly inhibited by an anti-CD9 mAb and several anti-CD81 mAbs, in both rodent and human *in vitro* models, by acting on the leukocyte side and on endothelial tetraspanins (59, 60). Accordingly, CD81 mAb (clone Eat2) administration reduced spinal cord inflammation *in vivo*, alleviating autoimmune encephalomyelitis (EAE) (59). Ly6C^+^ monocytes, which can derive in MoDCs (61), are key determinants for Th17 differentiation in the EAE mouse model (62, 63). Moreover, since ICAM-1 and VCAM-1 adhesion molecules are the ligands of leukocyte integrins Mac-1 (αMβ2) and LFA-1 (αLβ2) (for ICAM-1) and VLA-4 (α4β1) (for VCAM-1), we must emphasize the importance of endothelial tetraspanins as organizers of ICAM-1 and VCAM-1 containing docking structures during leukocyte extravasation (11, 64). Importantly, loss-of-function studies have demonstrated that CD81 is essential for cell rolling, arrest, and migration. In both monocytic cell lines and mouse primary splenocytes, CD81 facilitates rolling and arrest under shear flow, increasing the avidity of integrin VLA-4 (65) (Figure 2). The link between tetraspanins and MMP during immune cell migration has also been investigated. Bone marrow-derived macrophages (BMDMs) from CD9 and CD81 double deficient mice show reduced motility, through a mechanism dependent on the regulation of MMP-2 and MMP-9 expression and activity (Figure 2). Interestingly, CD81 and CD9 double deficient mice spontaneously develop pulmonary emphysema, with elevated numbers of alveolar macrophages and increased MMP activity (66). A similar increase in MMP-2 and MMP-9 production and activity was observed in BMDMs from CD9-deficient mice, which showed decreased macrophage motility with an increase in macrophage infiltration after intranasal administration of LPS (31).

It is important to mention that DC motility behavior depends on the environmental context. DC migration on two-dimensional (2D) surfaces, like endothelial cell surfaces of the circulatory system, require adhesive forces and integrin functionality; whereas migration in three-dimensional (3D) environments, as interstitial ECM, is ameboid and less adhesive, and largely driven by cytoskeletal deformability (67–69). Importantly, tetraspanins fine-tune DC migratory capabilities by tightly controlling Rac1 and RhoA spatio-temporal activation. CD81 controls the migration of MoDCs, by regulating the formation of lamellipodia, and the mobilization of preformed integrin clusters at the leading edge of migratory cells (70). This tetraspanin is essential for the formation of actin protrusions through a mechanism dependent on its interaction with the small GTPase Rac-1 (70, 71) (Figure 2). Integrin adhesiveness and lamellipodia formation are required for DCs migration on 2D surfaces, thus this kind of migration is impaired in the absence of CD81. However, CD81 is not required for DCs migration within 3D collagen scaffolds, corresponding with unaffected Rho-A activity (70) and pointing out the differential molecular requirements of DCs migration. CD37 also promotes Rac-1 activation, while CD82 inhibits RhoA (72). Consequently, CD37 deficient DCs have impaired migration from the skin to the draining lymph nodes *in vivo*, and reduced *ex vivo* DC migration in response to CCL19 (73). CD82 deficient DCs display the opposite phenotype (72). Absence of CD37 in BMDCs also reduces adhesion to fibronectin under low shear flow, and cell spreading (73), while CD82 deficiency increases DC spreading (72). Thus, CD37^hi^CD82^lo^ DCs would correspond to immature cells, showing increased migration and reduced capacity to activate naïve T cells, while CD37^lo^CD82^hi^ DCs would have an activated phenotype, being less motile and endowed with the proper presentation machinery to efficiently activate naïve T cells (72) (Figure 2). It is becoming increasingly clear that through the regulation of cytoskeletal rearrangement, integrins, and signaling molecules, tetraspanins constitute key players in APCs migration.

### MHC-II Trafficking and Antigen Presentation Take Place Within TEMs

**Figure 3:**
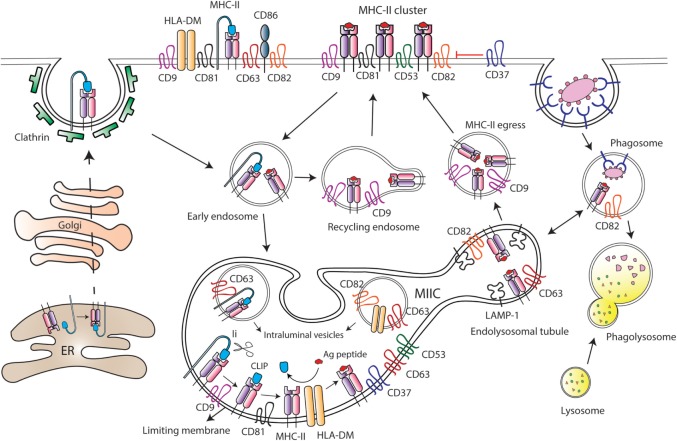
Tetraspanins regulate major histocompatibility complex class II (MHC-II) trafficking and surface expression at antigen-presenting cells (APCs). After synthesis, MHC-II molecules are transported to the plasma membrane of APCs in association with the chaperone Ii, which drives MHC-II internalization through clathrin-coated pits. Then, Ii is sequentially degraded by different proteases at the MHC-II-enriched endosomal compartment (MIIC), until MHC-II molecules remain bound only to the CLIP (class II-associated invariant chain peptide) fragment. Peptides derived from internalized antigens are subsequently loaded to MHC-II molecules with the help of the class-II-like chaperone HLA-DM. Several tetraspanins are greatly enriched at the MIIC compartment, and peptide-bound MHC-II molecules coupled to Ii are included into tetraspanin-enriched microdomains (TEMs). Tetraspanin CD9 is required for the efficient egress of MHC-II from MIIC to the cell surface, and it is essential for MHC-II endocytosis and recycling. CD82 together with CD63 accumulate in LAMP-1-enriched endolysosomal tubules that emanate from the MIIC. In MIIC intraluminal vesicles, CD63 is associated with MHC-II and HLA-DM; while CD82 only interacts with HLA-DM. In addition, CD82 is preferentially associated with peptide-bound MHC-II, and it is found in phagosomes prior to their fusion with lysosomes. At the APC surface, tetraspanins CD9, CD53, CD81, and CD82 are associated with MHC-II, and tetraspanin CD37 negatively regulates MHC-II clustering. Moreover, TEMs containing CD9, CD63, CD81, and CD82 include MHC-II, HLA-DM, and CD86 molecules.

Upon their arrival to the lymph nodes, DCs transfer the information collected at peripheral tissues to T lymphocytes triggering adaptive immune responses. This process of antigen presentation is mediated by MHC-II molecules, which are able to stably bind to antigenic peptides, and then present these fragments of exogenous proteins to effector T lymphocytes. MHC-II is expressed on professional APCs and associates with several tetraspanins, including CD9, CD37, CD53, CD81, and CD82, at the surface of APCs (74–76). It has been suggested that different tetraspanins may play a role in MHC-II clustering (Figure 3). CD37 negatively regulates MHC-II clustering, thus limiting antigen presentation by mouse splenic CD11c^+^ DCs. CD37 knock-out splenic DCs show increased T-cell stimulatory capacity, by a mechanism strictly dependent on peptide-bound MHC-II signals (77). CD81 and CD9 co-immunoprecipitate with I-A MHC-II molecules in mouse BM-derived DCs and B blasts, and I-A/I-E heterologous multimerization is reduced in CD9-deficient BM-derived DCs (78). However, functional analyses were not performed in the study of Unternaehrer and collaborators. Another study has also suggested that MHC-II, together with HLA-DM and CD86, was included in TEMs containing tetraspanins CD9, CD63, CD81, and CD82 (79). This study was performed using the CDw78 antibody, which recognizes a specific determinant on an MHC-II subpopulation. However, this biochemical analysis of MHC-II multimerization was performed using mild detergent conditions (CHAPS 1%). It was later demonstrated that CD9 and CD81 co-immunoprecipitation with MHC-II I-A/I-E multimers only occurs under these mild detergent conditions (80), not being observed when using more stringent conditions (Triton X-100). Thus, deficiency in CD9 or CD81 does not affect MHC-II clustering at the surface of mouse BM-derived DCs, while surface cholesterol content is essential for multimerization (80, 81). In addition, it was later demonstrated that the CDw78 determinant also recognizes peptide-bound MHC-II molecules coupled to the chaperone class-II associated invariant chain (Ii) (82). Intracellular trafficking of MHC-II molecules in APCs is a tightly regulated process, essential for proper antigen internalization, processing and subsequent presentation to T lymphocytes. Newly synthesized MHC II molecules associate with the chaperon Ii in the endoplasmic reticulum, which prevents premature peptide loading of MHC-II until MHCII-Ii complex enters the endocytic pathway (83) (Figure 3). The observation that peptide-bound MHC-II molecules coupled to the chaperone Ii (recognized by the CDw78 determinant) are included in TEMs (79, 82) suggests that tetraspanins could regulate MHC-II trafficking at the MHC Class II compartment (MIIC). Accordingly, there is considerable evidence supporting this hypothesis.

The MIIC is a multilamellar compartment that has similarities with late endosomes, being enriched in classical late endocytic markers, like LAMP-1, and in resident proteases, like cathepsins (84–86). Several tetraspanins, including CD37, CD53, CD63, CD81, and CD82, are highly enriched at the MIIC of human MoDCs and B cell lines (76, 87–89) (Figure 3). MHC-II diffusion rates are comparable to the diffusion values of CD63 and CD82, indicating inclusion into TEMs (89). Indeed, CD63 associates with MHC-II at both intraluminal vesicles and limiting membranes of the MIIC, and with the chaperone HLA-DM at the intraluminal vesicles. On the contrary, CD82 associates with HLA-DM at MIIC intraluminal vesicles and limiting membranes, but it only associates with MHC-II molecules at the limiting membrane (88, 89). CD82 would be mostly associated with peptide-bound MHC-II molecules, since it does not interact with MHC-II-coupled to Ii (88). Accordingly, CD82 and MHC-II are recruited together to phagosomes containing fungi or bacteria, before the fusion with lysosomes (90). Moreover, CD82 deficiency in DCs slightly reduces the maturation of MHC-II/peptide complexes (72). However, despite abundant evidence that tetraspanins dynamically interact with MHC-II and HLA-DM at the MIIC, they do not seem to be essential for peptide loading to MHC-II molecules. Downregulation of CD9, CD63, CD81, and CD82 in human cell lines does not affect surface expression of peptide-bound MHC-II (89), and CD9 deficiency in BMDCs does not affect antigen proteolysis (91). The dynamic interactions between these molecules at the MIIC compartment would rather indicate that TEMs are important organizers of MHC-II trafficking in APCs.

After being loaded with antigenic peptides, MHC-II molecules egress from the MIIC to the APC surface, a process that remains largely undefined. Recently, it has been reported that tetraspanin CD9 is important for MHC-II egress to the surface of mouse immature MoDCs (91). CD9-deficient MoDCs display increased accumulation of MHC-II molecules in acidic compartments, in which MHC-II colocalizes with LAMP-1. As a consequence, surface expression of MHC-II is decreased in the absence of CD9 (91). Upon DC maturation, tubular extensions emanate from the MIIC in a process dependent on microtubules and microtubule-adaptor proteins (92–94), thus transporting peptide-bound MHC-II molecules to the plasma membrane (95, 96). In mouse BMDCs stimulated with LPS, these dynamic tubular extensions are enriched in LAMP-1 and tetraspanins CD63 and CD82 and show accumulation of fluorescent OVA protein (93) (Figure 3). In mature MoDCs, CD9 is not involved in MHC-II egress from the MIIC to the plasma membrane, which would take place only in a CD9-independent manner (91). Therefore, transport of peptide-bound MHC-II to the cell surface might be dependent on different TEMs, whose composition would be tightly controlled before and after cell maturation.

After arriving at the APC plasma membrane, peptide-bound MHCII molecules are actively endocytosed and then recycled back to the surface *via* early endocytic compartments. MHC-II endocytosis occurs through clathrin- and dynamin-independent pathway(s) (83). Early studies suggested that in immature DCs, MHC-II internalization is facilitated through ubiquitination by the ubiquitin E3 ligase MARCH-I (97, 98). MHC-II ubiquitination would be less efficient in mature DCs due to reduced MARCH-I expression, which would result in an increase in MHC-II surface expression (98, 99). However, subsequent studies have challenged this view (100–102). MHC-II ubiquitination enhances the kinetics of degradation of peptide-bound MHC-II molecules in immature DCs (101) and prevents recycling of internalized molecules back to the membrane (102), without affecting endocytosis. MHC-II recycling back to the surface is highly increased upon DC maturation, greatly contributing to boost MHC-II surface expression (102). Other members of the MARCH family have been shown to be involved in tetraspanin turnover. CD81 is targeted to lysosomes in the presence of MARCH-IV and -VIII, but not MARCH-I. Accordingly, MARCH-IV downregulation by siRNA increases CD81 surface expression (103). The effect of MARCH proteins on CD81 turnover could also affect the expression levels of CD81-interacting partners included in TEMs. Importantly, a recent study showed that CD9 is essential for MHC-II endocytosis in both immature and mature MoDCs, by a mechanism independent on MHC-II ubiquitination. Moreover, CD9 deficiency prevents MHC-II recycling in mature MoDCs (91). Tetraspanins are therefore important players in MHC-II trafficking and surface expression at APCs.

Tetraspanins are relevant for antigen presentation to T lymphocytes. Early studies have suggested that disruption of TEMs by cholesterol depletion, which is an essential component of these microdomains (104), affects the capacity of APCs to stimulate T cell activation (79, 81). However, cholesterol depletion can also disrupt lipid rafts, which are also required for proper antigen presentation (105). More recently, the specific functions of individual tetraspanins during antigen presentation have been established. CD37 negatively regulates MHC-dependent antigen presentation to CD4^+^ and CD8^+^ T cells, while CD151 inhibits T-cell co-stimulation by mouse CD11c^+^ splenic DCs. As a consequence, mouse deficiency in those tetraspanins triggers CD4^+^ and CD8^+^ T-cell hyperstimulation (77). Sheng and collaborators have suggested that CD37 and CD151 could negatively regulate MHC clustering; however, despite the functional evidence demonstrated in their study, the molecular mechanisms behind CD37 and CD151 function remain to be determined. A similar phenotype was also observed with Tssc6^−/−^ and CD37^−/−^Tssc6^−/−^ mice, through a mechanism independent on DC costimulatory signals (106). CD63 knock-down in human B cell lines also enhances MHC-II-dependent CD4^+^ T cell stimulation, but in this case, the mechanism seems to be related with increased production of extracellular vesicles (107). In this sense, T cell activation can be induced by extracellular vesicles derived from mature DCs (18, 108), which are enriched in MHC-I and MHC-II, and several tetraspanins, like CD9, CD63, CD81, and CD82 (87, 109). MHC-II sorting into extracellular vesicles has been suggested to depend on its recruitment to TEMs (110–112). Together, these data suggest negative roles for some tetraspanins during antigen presentation. However, other tetraspanins can have the opposite effect. Indeed, mouse CD9^−/−^ MoDCs induce less CD4^+^ T-cell activation and proliferation than wild-type MoDCs, due to reduced surface expression of MHC-II (91). Strikingly, CD9-deficient Flt3L conventional DC (cDC) showed similar T-cell stimulatory capacity as wild-type cDCs, triggering comparable CD4^+^ T proliferation *in vivo* (91). The role of CD9 in antigen presentation seems therefore to be DC subset-specific, and it would be interesting to investigate the molecular mechanisms behind this difference. CD9 interacts with MHC-II, and engagement of this tetraspanin with antibodies promotes the formation of antigen-dependent conjugates between human CD14^+^ monocytes and T cells (113). CD9 could also play a role in antigen presentation through extracellular vesicles, since both are found at the MIIC and exosomes from mature splenic mouse DC lines (114). Together, these studies indicate that the strength of antigen presentation by professional APCs can be tightly regulated by TEM composition, with some tetraspanins playing positive roles while others limit T-cell activation signals.

### Tetraspanins Define Distinct DC Subsets

Dendritic cells can be classified in several subsets that differentially control the strength and duration of T-cell responses. The main populations that have been described are plasmacytoid DCs (pDCs) and cDCs, which can be divided into several subpopulations. Monocytes can also be precursors of different subsets of DCs found in different tissues in the steady state and can generate MoDCs during inflammatory reactions (61). Both human and mice individual DC subsets display different TEM composition (115). In addition, expression of specific tetraspanins can be modulated by DC differentiation and maturation. For instance, CD9 is differentially expressed on conventional and pDCs (115, 116).

Regarding cDCs, it has been suggested that they have a higher capacity to sense, process and present phagocytosed antigens to T cells than pDCs. cDCs are classified in two main subsets: CD141^+^ (BDCA3^+^) in humans and CD8α^+^ (CD11b^−^CD11c^+^) in mice; or CD1c^+^ (BDCA1^+^) in humans and CD4^+^ (CD11b^+^CD11c^+^) in mice (3, 4). Murine and human DC subsets have some similarities in their functional properties. In mice, CD8α^+^ cDC are found in lymphoid tissues and show similar phenotype and functional specialization to CD103^+^ cDCs, which are found in non-lymphoid organs. Both subsets express comparable levels of TLRs, CLRs, and chemokine receptors and have a higher capability to cross-present antigens to CD8^+^ T lymphocytes compared to CD11b^+^ DCs (3). Interestingly, CD141^+^ human and CD8α^+^ mouse cDCs show high expression of tetraspanins CD9, CD53, and CD81 (115), which associate with MHC-I (75, 117). CD141^+^ cDCs also display high levels of CD37, CD82, CD151, and Tspan31 (115). The other main subset of cDC is CD11b^+^ cDCs, which seem to be more efficient in MHC-II-dependent antigen presentation to CD4^+^ T lymphocytes, thus triggering polarization to Th2 and Th17 responses (3). The tetraspanin expression profile was somewhat variable when comparing CD1c^+^ human and CD11b^+^CD11c^+^ mouse cDCs (115). Indeed, CD1c^+^ human cDCs express very high levels of CD37, CD53, and CD81 and display intermediate to high levels of CD9, CD82, and CD151. In mice, CD4^+^CD11b^+^ cDCs show intermediate to low levels of CD9, CD53, CD81, and CD151 (115). As previously discussed, several of these tetraspanins are described to regulate different steps of MHC-II trafficking and antigen presentation by APCs. However, further studies are necessary to ascertain whether specific tetraspanin expression profiles can be used as markers of cDC subsets and/or define APC functions.

Plasmacytoid DCs, both in humans and mice, have the capacity to produce large amounts of type I interferons (IFN-α/β) in response to invading pathogens (118, 119). pDCs (BDCA2^+^ in humans, and B220^+^ in mice) are a small subset, and in mice express low levels of MHC-II, co-stimulatory molecules, integrin CD11c, and PRRs (119). Importantly, tetraspanins can be used as markers for the identification of different mouse and human pDC subpopulations. CD9 expression allows the recognition of immature and mature mouse pDCs subsets. CD9^+^Siglec-H^low^ pDCs have an immature phenotype, producing high levels of type I IFN and other pro-inflammatory cytokines. These cells are mainly present in mouse bone marrow and spleen, and when stimulated can induce strong CD4^+^ and CD8^+^ T cell responses *in vitro* and *in vivo* (120). In contrast, tissue resident pDCs are negative for CD9, do not produce IFN-α, and have a tolerogenic phenotype, increasing the numbers of Foxp3^+^CD4^+^ Treg cells in tumor-draining lymph nodes (120). Therefore, these two pDC subsets (CD9^+^ and CD9^−^) define cells at different maturation stages at steady state. Upon infection, cell activation would induce migration of CD9^+^ pDCs to the periphery, allowing the secretion of inflammatory cytokines at the infection site. Interestingly, upon maturation, CD9^+^ pDC upregulate markers of pDC differentiation but gradually lose CD9 expression (120). Distinct pDC mouse subsets can also be distinguished when looking at tetraspanin CD81. A small subpopulation of B220^+^CD5^+^CD81^+^ cells could be observed in blood, spleen, and bone marrow. This small subset does not produce IFN-α, while splenic CD5^−^CD81^−^ pDCs secrete high amounts of the cytokine (121). Similar CD81^−^ and CD81^+^ pDC subpopulations were observed in humans. Human pDCs are divided in two subsets depending on CD2 expression (122), and it has been recently demonstrated that CD2^high^ pDCs include CD2^hi^CD5^−^CD81^−^ and CD2^hi^CD5^+^CD81^+^ cells (121). Similarly to mice, human CD2^hi^CD5^+^CD81^+^ pDCs represent a relatively rare subpopulation that produce little or no IFN-α (121). This subset can, however, secrete other pro-inflammatory cytokines, like IL-12p40 and IL-6, and is capable of inducing B-cell proliferation and differentiation to plasma cells. In addition, CD2^hi^CD5^+^CD81^+^ pDCs are efficient inducers of CD4^+^ T cell proliferation and Treg differentiation (121). Interestingly, antibodies against CD81 and CD9, but not CD63, specifically inhibited IFN-α production by pDCs when co-cultured with HCV-infected hepatoma cells. This effect was specifically related to CD81 expression in pDCs and required Rac GTPase activity (123). Hence, the absence of tetraspanins CD9 and CD81 seems to identify small pDC subpopulations that do not produce type I IFN. However, whether these tetraspanin expression profiles define overlapping pDC subsets and/or if differential expression of tetraspanins is associated with specific APC phenotypes remain to be determined.

## Conclusion

In APCs, surface immune receptors and adhesion molecules, such as MHC molecules, co-receptors, PRRs, and integrins, associate with tetraspanins. Through the inclusion of these receptors in TEMs, tetraspanins can regulate their clustering, internalization, and intracellular trafficking, then affecting their downstream signaling. TEMs are thus important regulators of proper antigen uptake, processing and presentation. In addition, by modulating cytoskeleton-dependent processes, like outside-in integrin signaling, actin polymerization and cell spreading, tetraspanins are also key players in APC migration. Increasing evidence shows that different subsets of DCs having distinct requirements for antigen presentation and/or motility capabilities express specific repertoires of tetraspanins. This fine-tuned regulation warrants appropriate adaptive immune responses. Therefore, tetraspanins are potential targets for therapeutical interventions aiming to balance exaggerated immune responses in pathological inflammations and in immune-mediated chronic diseases.

## Author Contributions

MS and VR-P designed and wrote the review. VRP and FSM coordinated and edited the manuscript.

## Conflict of Interest Statement

The authors declare that the research was conducted in the absence of any commercial or financial relationships that could be construed as a potential conflict of interest.
